# Asthma and Allergic Diseases in Pregnancy: *A Review*

**DOI:** 10.1186/1939-4551-2-3-26

**Published:** 2009-03-15

**Authors:** Isabella Pali-Schöll, Cassim Motala, Erika Jensen-Jarolim

**Affiliations:** 1 Department of Pathophysiology, Center of Physiology, Pathophysiology and Immunology, Medical University of Vienna, Austria; 2 Division of Allergy, School of Child and Adolescent Health, Red Cross War Memorial Children's Hospital, Cape Town, South Africa

**Keywords:** allergy, atopy, newborn, pregnancy, prevention

## Abstract

Asthma and allergic disorders can affect the course and outcome of pregnancy. Pregnancy itself may also affect the course of asthma and related diseases. Optimal management of these disorders during pregnancy is vital to ensure the welfare of the mother and the baby.

Specific pharmacological agents for treatment of asthma or allergic diseases must be cautiously selected and are discussed here with respect to safety considerations in pregnancy. Although most drugs do not harm the fetus, this knowledge is incomplete. Any drug may carry a small risk that must be balanced against the benefits of keeping the mother and baby healthy. The goals and principles of management for acute and chronic asthma, rhinitis, and dermatologic disorders are the same during pregnancy as those for asthma in the general population.

Diagnosis of allergy during pregnancy should mainly consist of the patient's history and in vitro testing.

The assured and well-evaluated risk factors revealed for sensitization in mother and child are very limited, to date, and include alcohol consumption, exposure to tobacco smoke, maternal diet and diet of the newborn, drug usage, and insufficient exposure to environmental bacteria. Consequently, the recommendations for primary and secondary preventive measures are also very limited in number and verification.

## 

Allergy is a hypersensitivity reaction initiated by specific immunologic responses [1,2] against foreign, usually harmless, substances. The most common allergens worldwide include pollen, dust mites, molds, animal dander, cockroach, insect venom, and certain foods.

## Classification and Mechanism

Hypersensitivity against allergens can be mediated by either antibodies or T lymphocytes. Allergies or hyperreactivities have been classified by Gells and Coombs into types I through IV, with types I, II, and III being mediated by antibodies or immune complexes and type IV reactions as well as chronic stages of allergic diseases being mediated by T cells [1]. The focus of this review is set on asthma and type I allergy associated with pregnancy. The sensitization process involves the production of allergen-specific immunoglobulin E (IgE) antibodies, which are fixed to mast cells via their high-affinity receptor, Fc epsilon receptor I (Fc*ε*RI). On a subsequent encounter with the allergen, bridging of 2 or more IgE antibodies leads to degranulation of the mast cell with release of preformed mediators such as histamine, serotonin, tryptase, chymase, kininogenase, and heparin. Cross-linking is more likely if the allergen occurs in dimerized or multimerized form [3]. The mediator release results in the well-recognized acute allergic inflammation characterized by itching, redness, and tissue edema involving the skin, respiratory tract, circulation, and gastrointestinal tract. Immediate-phase type I allergic symptoms usually occur within several minutes after allergen contact. After degranulation, mast cells lose membrane area, become activated, and start a de novo synthesis of prostaglandins and leukotrienes from membrane arachidonic acid [4,5]. The newly synthesized cytokines and chemokines lead to the late-phase reaction associated with tissue edema by recruitment and activation of additional inflammatory cells, including basophils, eosinophils, and T helper type 2 (Th2) lymphocytes [6]. Late-phase reactions are observed several hours to days after allergen contact.

## Part 1: Specific Allergic Diseases in Pregnancy

### Epidemiology: Prevalence of Asthma and Allergy in Pregnant Women

Allergic sensitization to common allergens can be detected in approximately 25% to 35% of the general population in industrialized countries [7]. In the United States, about 18% to 30% of women in the childbearing age have allergic diseases, especially rhinitis [8] and asthma [9]. Other allergic diseases that may complicate pregnancy include conjunctivitis, acute urticaria, anaphylaxis, food allergy, and drug allergy. These disorders represent the most common group of medical conditions that complicate pregnancy. Asthma and allergic disorders can affect the course and outcome of the pregnancy. Pregnancy itself may also affect the course of asthma and other diseases [10]. Optimal management of these disorders during pregnancy is vital to ensure the welfare of the mother and the baby.

Specific pharmacological agents for treatment of asthma or allergic diseases must be cautiously selected. Although most drugs do not harm the fetus, this knowledge is incomplete. Any drug may carry a small risk that must be balanced against the benefits of keeping the mother and baby healthy.

### Safety of Asthma and Allergy Medication in Pregnancy

The ideal situation during pregnancy is "no pharmacological therapy," especially during the first trimester. However, the reality is that medications must be considered for pregnant patients with medical disorders, based on a thorough appreciation of the potential deleterious effects of untreated disease. For instance, women with asthma or allergic diseases require drug therapy during pregnancy to prevent symptoms severe enough to be life threatening to the mother or the fetus (eg, severe acute asthma that can result in hypoxia).

Of the available medications for allergic rhinitis (AR), chlorpheniramine is recommended as the first-generation antihistamine of choice for use during pregnancy [11]. Based on the information available (Table 1), loratadine or cetirizine may be considered the second-generation antihistamines of choice. The decision whether to use a first-generation or second-generation antihistamine for a particular patient must be individualized.

**Table 1:** Recommendations for Treatment of Asthma and Allergies in Pregnancy

Drugs preferred for use during pregnancy
Anti-inflammatory: cromolyn beclomethasone, prednisone
Bronchodilator: inhaled β_2_-adrenergic agonist, theophylline
Antihistamine: chlorpheniramine, tripelennamine
Decongestant: pseudoephedrine, oxymetazoline
Cough: guaifenesin, dextromethorphan
Antibiotic: amoxicillin
Drugs that generally should be avoided during pregnancy
α-Adrenergic compounds (other than pseudoephedrine)
Epinephrine (other than for anaphylaxis)
Iodides
Sulfonamides (in late pregnancy)
Tetracyclines
Quinolones

Adapted from the NAEPP expert group report [21].

Although most of the existing data regarding asthma and allergy medications during pregnancy have not demonstrated adverse effects, data regarding the use of oral corticosteroids have not been totally reassuring. In a recently published meta-analysis,[12] 6 cohort studies evaluating the relationship between first trimester maternal use of oral corticosteroids and congenital malformations did not show an increased risk of congenital malformations (summary odds ratio, 1.45; 95% confidence interval, 0.08-2.60). However, meta-analysis of the 4 case-control studies revealed a significantly increased risk of oral clefts in infants of corticosteroid-treated mothers (odds ratio, 3.35; confidence interval 1.97-5.69) [12]. Other adverse outcomes have also been attributed to oral corticosteroids, including an increased risk of preeclampsia,[13,14] preterm birth,[12,15] and lower birth weight [16]. However, the potential side effects of any drug must be balanced against the risks of the mother or the infant having inadequately treated disease. In the case of asthma, the risk of uncontrolled severe asthma (which may include maternal or fetal mortality) would usually be the greater risk, suggesting that oral corticosteroids must still be used when indicated for the management of severe gestational asthma.

### Asthma

Certain physiological changes occur normally during pregnancy (Table 2) [17]. These alterations are primarily the result of hormonal effects. They could potentially affect the course of asthma and predispose to hypoxia. The physiologically elevated position of the diaphragm and hyperventilation in pregnancy further increase the risk of hypoxia. Preexisting asthma symptoms may worsen, improve, or remain unchanged during pregnancy. Each of these 3 possibilities are observed in about one third of all cases [10].

**Table 2:** Normal Physiological Respiratory Changes During Pregnancy

Increased	Decreased
Tidal volume	Functional residual capacity
Minute ventilation	Residual volume
Alveolar-arterial O_2 _gradient	Diffusion capacity
Oxygen partial pressure	PaCO_2_
pH, normal or slightly elevated (respiratory alkalosis)	
Respiratory rate unchanged	

Adapted from Weinberger et al [17].

**Figure 1:**
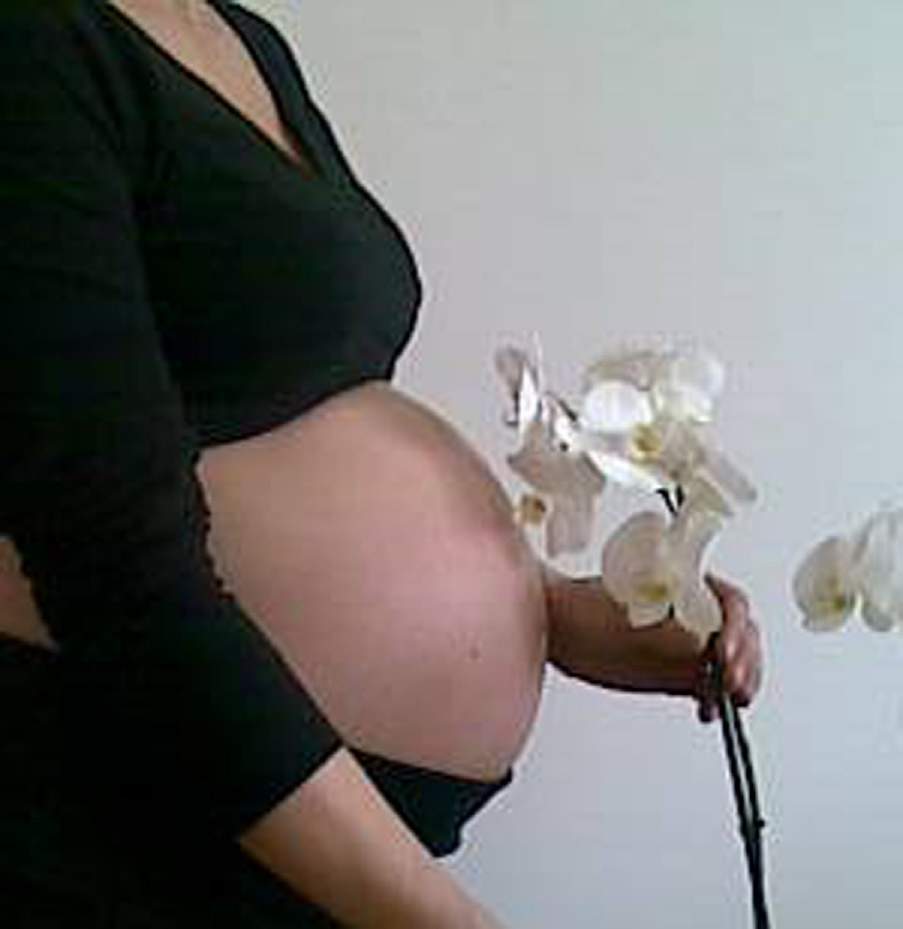
**Potential side effects of any drug taken during pregnancy by the mother must be balanced againts the risk of the mother of the infant suffering from inadequately treated symptoms**.

In general, patients with severe asthma are more likely to experience worsening of their disease during pregnancy. Alterations in maternal immunity, particularly a decrease in cell-mediated immunity, may predispose the pregnant asthmatic to infections and thus to acute exacerbations of asthma. Severe or poorly controlled asthma may be associated with an increased risk of fetal death, low birth weight, congenital malformations,[18] and parental complications [14]. Pregnant patients with severe or uncontrolled asthma should be considered high-risk and be treated promptly and optimally to ensure a favorable outcome for mother and baby. Thus, optimal asthma treatment is crucial, as the risk of preeclampsia, premature birth, low birth weight,[19] and maternal and neonatal hypoxia and morbidity [10,20] posed by undertreated asthma may be greater than that from the use of oral steroids for the treatment of asthma.

### Management of Chronic Asthma

The goals of management during pregnancy are the same as those for asthma in general, including prevention of severe exacerbations, improvement of quality of life (no interference with sleep or daily activities), and maintenance of normal lung function. Frequent regular follow-up visits to or by health professionals skilled in managing asthma are essential to ensure optimal success and safety of asthma management during pregnancy. The importance of effective 3-way communication among the patient, asthma specialist, and obstetrician cannot be overemphasized. Patients should have easy access to their physicians during times of increased symptoms. Education about asthma and its interaction with pregnancy reduces anxiety and improves compliance.

The recommendations for medical treatment have been summarized by a Working Group of the National Asthma Education and Prevention Program in a report on managing asthma during pregnancy [21]. A stepwise approach is suggested for medical treatment, where inhaled salbutamol is the preferred short-acting β agonist with an outstanding safety profile, and inhaled corticosteroids (eg, budesonide) should be used confidently as long-term control medications. Salmeterol is the preferred agent when long-acting β2 agonists are indicated in pregnant women as add-on treatment of persistent asthma. Leukotriene modifiers may be used as alternative add-on treatment; montelukast and zafirlukast are the preferred antileukotriene drugs (but zileuton is not).

Patients whose asthma is not controlled with maximal doses of bronchodilators and anti-inflammatory agents may need systemic corticosteroids. The lowest possible effective dose (alternate-day dose or single daily dose) should be used. Patients must be monitored closely for potential adverse effects of corticosteroids, especially gestational diabetes, preeclampsia, and intrauterine growth retardation.

### Management of Acute Asthma

Treatment of acute asthma is similar to that recommended for nonpregnant patients including inhaled β_2 _agonists, oxygen (essential), and corticosteroids (oral or parenteral) [22]. It is also reasonable to add nebulized ipratropium bromide in patients who do not respond to β_2 _agonists. Intravenous aminophylline is not generally recommended in the emergency management of acute asthma (because of its potentially harmful effects) but may be used in pregnant patients hospitalized for acute asthma (theophylline levels should be monitored). Criteria for admission and hospital management of the pregnant woman with acute asthma should be more lenient than for nonpregnant patients. Intravenous magnesium sulfate may be beneficial in acute severe asthma as an adjunct to inhaled β_2 _agonists and corticosteroids.

### Rhinitis

Significant nasal symptoms occur in approximately 30% of pregnant women [23]. Pregnancy-associated hormones have direct and indirect effects on nasal blood flow and mucous glands. The most common causes of nasal symptoms necessitating treatment during pregnancy are AR, rhinitis medicamentosa, sinusitis, and (non-AR) vasomotor [24]. *Vasomotor rhinitis of pregnancy *is a syndrome of nasal congestion and vasomotor instability limited to the gestational period. Allergic rhinitis is often preexisting but may occur or be recognized for the first time during pregnancy. Allergic rhinitis commonly coexists with asthma; 80% of asthmatic adults also have AR, and 20% to 50% of patients with AR also have asthma [25]. In a group of 1245 adult patients with documented asthma, 24% had seasonal AR only, 6% had perennial AR only, and 22% were considered to have both [26].

As with asthma, preexisting AR can worsen, improve, or remain unchanged during pregnancy [27]. Furthermore, during pregnancy, nasal congestion can worsen, although the exact mechanism for this is not defined.

The general principles of treatment of pregnant women with asthma [21,22,28] and AR [29] do not differ from the stepwise approach recommended for treatment of nonpregnant women. Intranasal cromolyn, intranasal steroids, and montelukast are the preferred drugs for the treatment of rhinitis because of the low risk of systemic effects. Second-generation antihistamines such as loratadine or cetirizine [21,29] can also be used (after the first trimester--as a general precaution). Adjunctive treatment of rhinitis, which is permitted in pregnancy, includes oxymetazoline drops or spray for nasal congestion, pseudoephedrine (after the first trimester) for persistent nasal congestion, and buffered saline sprays for nasal dryness, nasal bleeding, and vascular congestion associated with pregnancy.

### Anaphylaxis

The exact prevalence of anaphylaxis during pregnancy is unknown, but it is extremely uncommon [30]. The fetus seems to be relatively protected from anaphylaxis perhaps because the placenta does not transmit specific IgE antibodies to the fetus [31]. However, maternal hypoxia or hypotension associated with anaphylaxis may be catastrophic not only to the mother but also to her fetus. Maternal anaphylaxis has been associated with fetal distress, brain injury, and fetal loss (presumably because of diminished uteroplacental perfusion), as well as neonatal death [32-35]. Any agent that can cause anaphylaxis in the nonpregnant state could also cause anaphylaxis in the susceptible or sensitized pregnant patient. Even breast-feeding has been associated with anaphylaxis in 2 postpartum women. The reactions occurred within 1 to 3 days postpartum and resolved within 1 to 2 days. Changing levels of progesterone and triggering by nonsteroidal anti-inflammatory drugs were suggested as potential causes [36].

The management of anaphylaxis during pregnancy is the same as for nonpregnant patients. Of the routine antianaphylaxis medications, epinephrine and diphenhydramine have been implicated in causing fetal malformations. However, the tentative nature of these data and the lack of equally effective substitutes justify their use in pregnancy for this life-threatening emergency. Adequate intravascular volume repletion and oxygenation are particularly important in the management of anaphylaxis during pregnancy to prevent both maternal and fetal complications. The pregnant hypotensive patient should be placed on her left side to prevent added positional hypotension resulting from compression of the inferior vena cava by the gravid uterus [37]. Intravenous epinephrine may be required despite its potential to cause decreased uteroplacental blood flow. Glucocorticoids should be administered early to patients with severe anaphylaxis. For laryngeal spasm, intubation and, in rare cases, tracheotomy may be necessary.

### Adverse Reactions to Food

No studies have focused explicitly on the prevalence of food allergy in pregnant women primarily because often the immune response in the offspring is the main focus of research. A cohort study on the Isle of Wight, United Kingdom, found adverse reactions to food in approximately 20% of 969 pregnant women [38]. The results of this observational study were based on questionnaires filled out by the mothers after childbirth. From murine studies, it may be expected that the sensitization of a mother to a (food) allergen will also bias the immune response of the offspring toward Th2 [39,40].

### Atopic Eczema (Dermatitis)

Gestational itchy dermatoses are relatively common, with eczema being diagnosed in 30% to 50% of all cases [41]. However, the exact prevalence of allergic forms of dermatitis in the pregnant population is not known. The importance of an exacerbation of eczema and dermatitis during pregnancy should not be underestimated as readout of the atopic state, being of interest for the genetic predisposition of the newborn [42]. Most women with atopic dermatitis have lived with their condition long before becoming pregnant. The course is variable [43].

Treatment of atopic dermatitis during pregnancy should emphasize avoidance of triggering factors and reliance on topical treatment in an attempt to reduce dryness and pruritus, modulate inflammation, and treat secondary infections. Oral antihistamines should be avoided during the first trimester, unless definitely clinically indicated, and then should be used at the lowest effective dose, starting with chlorpheniramine, loratadine, or cetirizine. Topical steroids should be selected based on potency considerations, given the potential for systemic absorption and increased skin surface area of pregnancy. Severe intrauterine growth retardation occurred in the infant of a mother who applied 40 mg/d of topical triamcinolone cream from week 12 to 29 of gestation to treat her atopic dermatitis [44]. Topical corticosteroid treatment should be initiated when clinically indicated, with the least potent preparations such as hydrocortisone (0.5% to 1%), reserving more potent preparations for more recalcitrant areas in selected patients. The recently introduced topical calcineurin inhibitors (pimecrolimus and tacrolimus), although effective in the treatment of atopic eczema, are not recommended for use in pregnant patients because of the lack of safety data.

### Urticaria and Angioedema

The pattern and causes of urticaria and angioedema in pregnancy are similar to those in nonpregnant patients. A unique form of urticaria associated with pregnancy, which tends to recur in subsequent pregnancies (*pregnancy urticaria*), has been reported [45]. The pathogenesis of this condition is unknown, although there is speculation that it may be caused by allergic sensitization to endogenous hormones [46,47]. The first step in treatment of urticaria and angioedema in pregnancy is identification and avoidance of causative factors. Antihistamines should be avoided if possible, but if required, chlorpheniramine, loratadine, or cetirizine may be used. Rarely, systemic corticoids may be used for severe recalcitrant urticaria and angioedema during pregnancy.

### Drug Allergy

There is a lack of data on the prevalence of adverse drug reactions during pregnancy. The diagnosis and treatment of drug allergy are the same in pregnant as in nonpregnant patients.

### Diagnosis of Allergy During Pregnancy

The diagnosis of allergy in pregnant women should focus on a detailed medical history and symptom analysis. For diagnosis, a diary of allergy symptoms and avoidance of specific allergens accompanied by monitoring of changes of allergic symptoms may be helpful. However, it is important not to put the mother on a rigid elimination diet because this could negatively influence the nutrition of both the mother and the growing infant [48].

In vitro diagnostic tools such as serological testing, for example, radioallergosorbent test for type I allergy or the lymphocyte transformation test for type IV allergy [49] are preferred to in vivo testing. Although not contraindicated, skin prick testing should be postponed until after birth because of possible, although rare, anaphylactic reactions to skin prick testing. The same applies to in vivo tests such as food challenge tests and patch tests. Only in situations where in vitro diagnosis is not conclusive and the mother is at risk for developing allergy symptoms should in vivo skin tests be used. Examples include the occurrence of generalized eczema requiring identification of the cause before treatment or a suspected allergy to a drug needed for therapy of the mother (eg, penicillin for treating syphilis) [50]. Importantly, when performing in vivo testing such as skin tests or provocation test, the exceptional immunologic status accompanying pregnancy should be considered when interpreting test results. Any unexpected test result and any symptoms that change over time should be reevaluated after pregnancy. In addition, the attending physician should bear in mind that some symptoms may be a direct result of pregnancy and not allergy related, for example, vasomotor rhinitis in the last trimester [51] and gestational urticaria (pruritic urticarial papules and plaques in pregnancy) [52].

### Nonpharmacological Management of Allergic Diseases During Pregnancy

Mothers with diagnosed allergy should avoid exposure to and contact with specific allergens (including food allergens) and should especially avoid the inhalation of potent triggers for asthma, such as animal dander, house dust, tobacco smoke, and irritating pollutants [22].

Hyposensitization (immunotherapy), which may be selectively indicated in certain patients with AR, bee venom sensitivity, and asthma should not be initiated during pregnancy because of the risk of systemic reactions. For patients who were already on immunotherapy before the pregnancy, maintenance treatment may be continued safely during pregnancy. However, the allergen dose should not be increased during pregnancy but rather reduced if necessary [29].

### Summary

Many women experience type I allergies during pregnancy. Allergy diagnosis during pregnancy should preferentially consist of recording the patient's history for precise recommendation of allergen avoidance and in vitro testing only. In vivo testing should generally be postponed until after pregnancy, unless diagnosis for therapeutic intervention is urgently needed. In general, treatment of asthma and allergic diseases does not differ from that in nonpregnant women. Immunotherapy may be continued as maintenance treatment but should not be initiated during pregnancy.

## Part 2: Prevention of Asthma and Allergies in Early Life

### Infants With High-Risk of Allergy

The potential risk for the development of atopy among newborns is high. Genetic components and complex environmental factors contribute to the etiology of asthma and atopic diseases. To date, there are no biomarkers that can reliably predict which newborn will develop atopy. However, an atopic family history has been recognized to be a simple and inexpensive risk indicator. The degree of risk seems to be directly related to the family history of allergy and especially to maternal atopy,[53] particularly when the mother has diagnosed atopic eczema and has elevated IgE levels. If neither parent is allergic, the chance for allergies in the child is about 15%. If 1 parent is allergic, the risk increases to 30%, and if both are allergic, the risk is greater than 60%, especially for developing the same organ-specific symptoms [54]. The influence of maternal sensitization on the immune response of newborns was recently shown in a human observational study of AR, in which only the organ-specific symptoms of the mother but not the father were demonstrated to be relevant for the imprinting of the child [55]. However, sensitization may not be restricted to the organ at which the mother experiences allergic symptoms because asthma or wheezing in children was associated not only with asthma and AR, but also with eczema or any allergic disease in their parents and siblings [56,57].

### Transfer Mechanisms of Allergy (Immunopathogenesis of Atopy)

The allergic status of the mother can potentially affect the immune response of the offspring through numerous factors that are transferable via the placenta or breast milk. The direct transfer of food or inhalant allergens via the placenta or breast milk has long been recognized [58-60]. In addition, antibodies can be transferred to the child via placenta (IgG, IgA) or breast milk (IgA, IgG, IgM, IgE),[61,62] and even a transamniotic transfer of intact maternal IgE into the amniotic fluid can occur [63].

Only a very limited number of human studies on the maternofetal transfer of cytokines and chemokines are available. For instance, interferon-γ (IFN-γ) and interleukin-6 (IL-6) were detected in the colostrums of healthy women [64]. The transfer of maternal cytokines was confirmed in a study of suckling piglets [65]. In vivo and in vitro models have shown that such transfer may lead to reduced neonatal immunity. Also chemokines, for example, IL-8, a cytokine that is regulated on activation with normal T cell expressed and secreted (RANTES), IFN-γ -inducible protein 10, and monokine induced by IFN-γ have been detected in breast milk [66,67]. However, cytokines and other soluble inflammation markers are found in breast milk from atopic as well as in nonatopic women.

Another factor that should be considered is the composition of fatty acids present in breast milk, which may influence the immune response of the child [68].

In addition to the previously mentioned soluble factors, also cells are transferred in utero, for instance, leukocytes pass from mother to fetus. This seems to be important because allergen-specific T cells transferred from 1 mouse mother to another can transmit asthma risk to the offspring of the recipient mouse mother [69]. Trafficking of cells could therefore be responsible for the passing of allergy risk from mother to fetus.

### Environmental Risk Factors for Atopy

The causes of allergy in general and of specific sensitization in newborns in particular have not yet been completely determined. Besides the role of genetic predisposition, some factors have been identified that may either contribute to sensitization of the mother and to the subsequent transfer of a predisposition for allergy to the offspring, or that directly induce sensitization in offspring that manifests shortly after birth or at a young age.

### Exposure to Tobacco Smoke

One of the most frequently discussed risk factors for induction of sensitization in all populations and age groups is tobacco smoke (active smoking or the passive environmental exposure). In a recent mouse study, exposure to smoke in utero induced a higher risk of sensitization against allergens in adult age in the offspring [70]. Accordingly, in human blood samples, Th2 cytokines responsible for a predisposition toward allergy were elevated in the neonates only of mothers who had smoked during pregnancy [71]. In addition, total and specific IgE levels,[72,73] total eosinophil counts,[74] incidence of airway disease,[74] and positive results on skin prick tests [75] were also increased in children who were exposed to smoke either during pregnancy or in early childhood. Alarmingly, in the birth cohort study from the Isle of Wight, about 43% of children had been exposed to environmental tobacco smoke during their first year of life [38].

### Alcohol Consumption

Alcohol consumption by the mother during pregnancy is associated with higher total IgE levels in cord blood [76]. Alcohol consumption in adults is also a risk factor for elevated specific IgE levels against food antigens [77,78].

### Maternal Diet

The maternal dietary component consumed during gestation could influence the immune status of the child. For instance, different polyunsaturated fatty acids (PUFAs) have been shown to differently influence the outcome of eczema in the child. A diet higher in n-6 PUFAs--as present, for example, in margarine and vegetable oils--seems to be more likely to induce eczema than n-3 PUFAs, which are for instance found in fish [79]. Accordingly, another study showed that fish consumption decreased eczema [80]. However, it may not be the absolute content but the ratio of n-6 to n-3 PUFAs that may influence the development of either tolerance or sensitization to food, as a high ratio of 9 of n-6/n-3 in the diet of the mother prevented the induction of oral tolerance to ovalbumin in the offspring in a rat study [81]. Celery and citrus fruits seem to increase the risk of food sensitization, whereas vegetable oils, raw sweet pepper, and again citrus fruit increase sensitization to respiratory antigens [79]. Interestingly, apples consumed during pregnancy were able to decrease wheezing in children [80].

According to a directive of the Commission of European Communities from 2005, the most allergenic foods have to be labeled because of their potency of eliciting severe allergic reactions: these are crustacean, fish, nuts, milk, egg, wheat/gluten, peanuts, soy, sesame, mustard, and celery. It has long been proposed that the mother should avoid such foods containing potential allergens during pregnancy and lactation to prevent food sensitization in the child. However, recent studies suggest that allergen exposure may be necessary to induce tolerance, and moreover, a balanced diet prevents malnutrition of both mother and child [82]. Furthermore, alterations of the maternal diet, that is, avoidance of milk and egg consumption during pregnancy did not seem to lower the risk of sensitization in the child [83,84].

### Use of Antiacid Medications

The changing hormone levels during pregnancy lead to a lower esophageal sphincter pressure in the mother, and concomitantly with growing volume of the fetus, these factors often result in heartburn, reflux, and abdominal pain. Studies found that about 70% of pregnant women are affected by these symptoms during their last trimenon,[85] and 50% of them are likely to take acid-suppressing medication. However, recent animal and human studies indicate that acid suppression and the resulting elevated pH in the stomach may lead to an increased risk of sensitization to food [86,87]. This sensitization of the mother was shown to lead to an increased risk of food allergy in the newborn in a BALB/c mouse model [39]. As previously discussed, an allergic status of the mother has the potency to affect the immune response of the offspring by numerous factors transferable via placenta or breast milk and even via the transamniotic route, for instance, intact maternal IgE in amniotic fluid, maternal DNA in cord blood, leukocytes, and chemokines such as IL-8, RANTES, interferon-γ-inducible protein 10, or monokine induced by IFN-γ, allergens, as well as antibodies. Furthermore, the offspring of sensitized mothers are prone to experience suppression and a later onset of normal levels of the Th1 cytokine IFN-γ because of a lower frequency of IFN-γ-producing cells, and therefore a further bias toward a Th2 immune response.

### Infant's Diet

Regarding the nutrition of the baby, reduced breast-feeding and early introduction of solid food have been discussed as confounders to allergy development. However, a randomized trial revealed quite contrary that promoted and prolonged (exclusive) breast-feeding is not able to prevent development of allergy or asthma in children at the age of about 6 years [88]. In addition, a systematic review of several studies found no clear negative association between early solid food introduction and the development of asthma, food allergy, AR, or animal dander allergy [89].

### Prematurity and Low Birth Weight

As shown within the Manitoba birth cohort study, prematurity and low birth weight are not associated with an increased risk for development of food allergy in childhood [90]. The impact of these 2 factors for sensitization to other allergens, such as aeroallergens, has not been investigated. However, 1 study showed that adolescents who had been born prematurely had a substantially decreased expiratory volume and increased bronchial hyperresponsiveness, making them potential candidates for developing asthma [91].

### Birth by Cesarean Delivery

The delivery of a baby by cesarean delivery has been suggested to be associated with increased risk of allergies and atopic diseases in the child, most probably because of a lower bacterial exposure for the child. In a recent population-based cohort study in Norway, it was shown that a positive association exists for cesarean delivery and risk of asthma in the child (especially when the cesarean delivery was performed because of an emergency in contrast to a planned section) [92]. Bager and colleagues [93,94] also showed an increased risk for asthma, allergic rhinitis, and possibly for food allergies in their meta-analysis. However, because of the low numbers of an allergic outcome associated to cesarean delivery (only 1%-4%), the authors concluded that the epidemic increase of allergic diseases might not be attributable to the increased numbers of cesarean delivery.

### Insufficient Exposure to Environmental Bacteria

The *hygiene hypothesis *states that low exposure of the mother during pregnancy and of the newborn in early life to environmental bacteria contributes to a Th2-biased immune response. This hypothesis has been confirmed by several experimental animal and epidemiological human studies (reviewed in Renz et al [95]).

### Preventive Measures for Mother and Child

Strategies for prevention of atopic diseases may be categorized as primary, secondary, and tertiary. Primary prevention addresses symptom-free children at risk (ie, without the established disease). Secondary prevention addresses individuals with early indicators of atopic disease. Tertiary prevention is directed at patients with a chronic disease to prevent additional problems related to the disease.

Primary prevention has been suggested for those newborns who have at least 1 parent or sibling with proven atopy. However, it must be borne in mind that a proportion of children who develop atopic manifestations in the first years of life come from families in which neither parents nor siblings are atopic. In addition, a significant percentage of infants from families with an atopic background do not develop atopy during childhood. This suggests the possibility that some infants who would never have developed atopy would undergo preventive regimens, whereas others who become symptomatic in early life would not have received adequate advice. This review will deal only with dietary and environmental control measures (see later) for the prevention (primary and secondary) of atopic diseases. Preventive pharmacotherapy and immunotherapy will not be discussed.

### Smoking

Maternal smoking and passive exposure to smoke should both be avoided. This is especially important for pregnant asthmatic patients, in whom smoking-related morbidity is independent of--and adds to--the morbidity resulting from asthma [96]. Although there are contradicting epidemiological and experimental results regarding the direct influence of smoking on total and specific IgE production,[97,98] smoking should nevertheless be avoided for obvious reasons, such as carcinogenic smoke constituents and the vasoconstrictive effect of nicotine.

### Alcohol

Alcohol intake should be avoided by the pregnant mother because of well-known toxic effects on both mother and fetus. Moreover, alcohol consumption has been shown to induce higher IgE levels against aeroallergens in atopic patients [76].

### Maternal Diet During Pregnancy

A Cochrane database meta-analysis of 4 clinical studies concluded that antigen avoidance during pregnancy is unlikely to reduce the child's risk of developing atopic disease, and dietary restrictions could adversely affect maternal or fetal nutrition [99]. Although previously suggested that pregnant women with risks avoid peanuts, a subsequent study found no evidence of prenatal sensitization from maternal consumption of peanuts [100]. In addition, recent publications revealed that an effect (positive or negative) of peanut avoidance on sensitization was not detectable (although too few valuable subjects and the observational nature of the reports reduce the ability to make firm conclusions) [101,102]. Generally, there is a lack of evidence for maternal pregnancy restriction diets. Additional recent studies about pregnancy diets reveal that components other than specific allergens, such as fat, may influence atopy outcomes [103,104]. Therefore, no special diet for the mother is required during pregnancy. On the contrary, the diet should be well balanced and consist of as many different nutrients as possible, as the current literature suggests that antigens obtained via the oral route during pregnancy and lactation are needed to develop allergen tolerance in the child [105]. In line with this, there was no association between maternal intake of foods during pregnancy and the occurrence of asthma, respiratory disease, or allergy in 5-year-old children [80]. Furthermore, many studies found no benefit of a restricted diet avoiding the consumption of allergy-inducing food during pregnancy [82].

However, if the mother is already allergic or if there is a family history of atopy or allergy, food comprising potential allergens should be avoided. In such instances, the avoidance of peanuts, nuts, fish, eggs, and sesame during the last 3 months of pregnancy may have a protective effect [106]. In this respect, government recommendations must be carefully worded, as 1 study found that avoidance of peanuts was overdone among pregnant women: although such avoidance was recommended only for women with atopy risk, 65% of all pregnant women had avoided peanuts during pregnancy (42% of all women who responded had heard about the advice, eg, from their midwives, and 50% subsequently changed their diet) [101].

Numerous individual nutrients consumed by the mother during pregnancy have been suggested as being preventive for development of atopic diseases in the child; these include apples or fish,[80] vitamin D,[107] vitamin E,[108] and various probiotics, the latter having been shown to be effective in mouse models [109] and human studies [110].

### Antiacids During Pregnancy

Recent studies have indicated that acid-suppressing medications can promote sensitization in adults [86,87]. Therefore, it is suggested that antiacids should be taken only when prescribed by an attending physician to the childbearing mother. Treatment of reflux during pregnancy should focus on nonpharmacological measures, such as avoiding large meals shortly before bedtime, sleeping with the upper body elevated, and avoiding extensive consumption of coffee, sweets, and fatty foods. In addition, smoking is a cause of reflux and should be avoided. If these measures are unsuccessful, then prescription of antiacid drugs should be considered, for example, in the form of a step-up program beginning with antacids, and in case of failure with histamine-2 receptor antagonists, whereas proton pump inhibitors should only be used in women with intractable symptoms or complicated reflux disease [111,112].

### Avoidance of Other Allergens During Pregnancy

Avoidance of other allergens is necessary only if the pregnant patients are already sensitized (eg, to cats, dogs, horses, or house dust). However, allergen avoidance may not be preventive or practical in all cohorts [113].

### Maternal Diet During Lactation

Regarding maternal lactation diets, a Cochrane database meta-analysis [99] found some evidence of reduced atopic dermatitis, but suggested that more studies are needed. A previous expert review that was not a structured meta-analysis and did not exclude as many studies as the Cochrane review concluded that special maternal lactation avoidance diets were unnecessary [114]. During lactation, a special diet for the mother (or, if not breast-feeding, hypoallergenic formula nutrition for the infant) should be considered only in individuals with an established atopy risk or existing allergy [82,115].

### Diet of the Infants

As mentioned previously, for breast-feeding, there is insufficient evidence to determine whether it is more protective against allergies and asthma than bottle feeding because in a recent study, prolonged and exclusive breast-feeding could not decrease the rate of allergies or asthma in children [88]. Furthermore, there are data supporting that breast-feeding should not be continued beyond 9 months of age because doing so seems to increase the child's risk for atopic diseases, such as atopic dermatitis and food hypersensitivity [116].

Some studies investigated the effect of reduced exposure of infants to allergen-provoking food. In cases where a reduced sensitization to food was found, this protective effect was short-lived and was primarily observed during the first year of life.

As a further preventive measure, the early introduction of solid food (ie, baby food from jars and table food) into the infant's diet has to be revisited. It has been suggested that foods containing allergy-provoking proteins such as milk, egg, peanuts, tree nuts, fish, and seafood should not be introduced into the diet of the child before the age of 6 months to 3 years [117]. However, contact with and exposure to antigens may be necessary in early life to develop tolerance in the immune system [118]. This process is active and specific, much like the process of sensitization [119]. Thus far, studies on delayed introduction of solids led to conflicting results.

### Summary

Based on currently available evidence, guidelines for primary and secondary prevention of allergic disease can be summarized as follows (Table 3).

**Table 3:** Primary Prevention of Asthma and Allergies in Early Life

**Preventive Measures**

Smoking and exposure to environmental tobacco smoke should be avoided, especially during pregnancy and early childhood
Alcohol consumption should be avoided by the mother during pregnancy and lactation
Damp housing conditions should be avoided and indoor air pollutants reduced, especially for high-risk children (history of atopy or allergy in a first-degree relative)
Breast-feeding should be performed exclusively until 6 months with no special diet for the lactating mother (except when the mother is already diagnosed with food allergy)
Nonprescription drugs should be avoided during pregnancy and lactation unless recommended by a physician

### Smoking and Alcohol

Smoking and passive exposure to cigarette smoke as well as alcohol consumption should be strictly avoided.

### Diet

There should be no special diet for the mother during pregnancy and lactation, unless the mother or child has a diagnosed food sensitization. In infants with risk of allergy, introduction of solid foods in general should be postponed until 6 months of age, milk products until 12 months, hen's egg until 24 months, and peanut, tree nuts, fish, and seafood until at least 36 months.

### Other Allergens

Avoidance of allergens (pets, house dust, contact allergens, drugs) is not recommended except when sensitization has already been diagnosed.

### Reflux Treatment

Pregnancy-associated reflux should be treated by nonpharmacological measures first.

### Atopy

For newborns at suspected risk for atopy, that is, with a history of atopy/allergy in a first-degree relative (parents, siblings), exposure to irritating air pollutants and airborne allergens such as molds should be minimized.

### Breast-feeding

Infants should be breast-fed for at least 4 months but no longer than 9 months. Special hypoallergenic formula (extensively hydrolyzed, not soy-based) should be used only if the child is diagnosed with atopy.

### Drugs

All nonprescription drugs (eg, antiacids) should be avoided during pregnancy and lactation unless recommended by a physician; patients should avoid intake of any medication including over-the-counter substances without consulting their physicians.

For more information on primary, secondary, and tertiary prevention of allergy, readers are referred to the document, "Prevention of Allergy and Allergic Asthma," an article based on findings presented at the World Health Organization/World Allergy Organization meeting in January 2002 [120]. This document also includes a summary of evidence-based guidelines and strength of recommendations.

## End Note

Research for this article was supported by Hertha Firnberg stipend T283-B13 and SFB F1808-B13 from the Austrian Science Fund (FWF). A synopsis from this review is also submitted for publication on the WAO homepage (http://www.worldallergy.org/adrc/). We would like to acknowledge Gefried Pali for the photograph arts.
